# Exogenous 5-Aminolevulenic Acid Promotes Seed Germination in *Elymus nutans* against Oxidative Damage Induced by Cold Stress

**DOI:** 10.1371/journal.pone.0107152

**Published:** 2014-09-10

**Authors:** Juanjuan Fu, Yongfang Sun, Xitong Chu, Yuefei Xu, Tianming Hu

**Affiliations:** Department of Grassland Science, College of Animal Science and Technology, Northwest A&F University, Yangling, Shaanxi Province, P. R. China; Chinese Academy of Sciences, China

## Abstract

The protective effects of 5-aminolevulenic acid (ALA) on germination of *Elymus nutans* Griseb. seeds under cold stress were investigated. Seeds of *E. nutans* (Damxung, DX and Zhengdao, ZD) were pre-soaked with various concentrations (0, 0.1, 0.5, 1, 5, 10 and 25 mg l^−1^) of ALA for 24 h before germination under cold stress (5°C). Seeds of ZD were more susceptible to cold stress than DX seeds. Both seeds treated with ALA at low concentrations (0.1–1 mg l^−1^) had higher final germination percentage (FGP) and dry weight at 5°C than non-ALA-treated seeds, whereas exposure to higher ALA concentrations (5–25 mg l^−1^) brought about a dose dependent decrease. The highest FGP and dry weight of germinating seeds were obtained from seeds pre-soaked with 1 mg l^−1^ ALA. After 5 d of cold stress, pretreatment with ALA provided significant protection against cold stress in the germinating seeds, significantly enhancing seed respiration rate and ATP synthesis. ALA pre-treatment also increased reduced glutathione (GSH), ascorbic acid (AsA), total glutathione, and total ascorbate concentrations, and the activities of superoxide dismutase (SOD), catalase (CAT), ascorbate peroxidase (APX) and glutathione reductase (GR), whereas decreased the contents of malondialdehyde (MDA) and hydrogen peroxide (H_2_O_2_), and superoxide radical (O_2_^•−^) release in both germinating seeds under cold stress. In addition, application of ALA increased H^+^-ATPase activity and endogenous ALA concentration compared with cold stress alone. Results indicate that ALA considered as an endogenous plant growth regulator could effectively protect *E. nutans* seeds from cold-induced oxidative damage during germination without any adverse effect.

## Introduction

Cold stress is commonly defined as the low temperature range that is adequate to alter growth without stopping cellular processes [1]. Cold greatly influences seed germination, and consequently induces a reduction in germination rate and a delay in the initiation of the germination and seedling establishment [2]. Thus, it is worthwhile to clarify the physiological mechanisms of poor seed germination caused by cold stress and to develop reasonable strategies to alleviate the adverse effects of cold on seed germination thereby plants establishment on low temperature environment, especially at high altitude.

Cold is one of severe environmental stresses that disrupts the metabolic balance of cells, resulting in membrane damage [3], reduction of cellular respiration [4], and production of reactive oxygen species (ROS) [5]. In plants, the antioxidant enzymes are important defense systems to detoxify ROS [6]. ROS scavenging enzymes in plants include superoxide dismutase (SOD), peroxidases (POD), catalase (CAT), guaiacol peroxidase (GPX), ascorbate peroxidase (APX), dehydroascorbate reductase (DHAR) and glutathione reductase (GR) [7], [8]. A large body of evidence has demonstrated that the antioxidant systems play important roles in protecting plants against oxidative damage induced by cold stress [3], [9].

The 5-aminolevulenic acid (ALA) is a key precursor in the biosynthesis of all porphyrins compounds such as chlorophyll, heme and phytochrome [10]. A number of reports show that exogenous ALA improves the growth and yield of a number of plants by enhancing chlorophyll contents and the rate of photosynthesis [11], [12]. It is also known that ALA in low concentration regulates key physiological processes associated with plant growth under various abiotic and biotic stresses, including low or high temperature [7], [13], salinity [14], drought [15] and heavy metals [16]. In contrast, high levels of ALA can promote enhanced production of ROS, thereby enhancing oxidative stress in plants [17]. These results suggest that ALA has a great application potential in agricultural production as a new non-toxic endogenous substance [18].

*Elymus nutans* Griseb., a perennial cool-season grass, is distributed in the north, northwest and southwest regions, especially on the Qinghai-Tibetan Plateau from 3,000 to 5,000 m in China [19]. *E. nutans* has been traditionally used as typical native forage and has often been collected and dried as long cool season [20]. Recently, it has been widely planted in cultivated pastures in alpine areas, owing to its high adaptability, good nutrition, high yield and good resistance to cold, drought and biotic stress [19]. Thus, an investigation of seed germination in low temperature is important to wild *E. nutans* establishment at high altitude in Qinghai-Tibetan plateau. Chen and Jia [20] reported *E. nutans* also plays a pivotal role in animal husbandry and environmental sustenance in China. However, to date, no specific information is available regarding the effects of ALA on cold stress resistance of *E. nutans* seeds. Moreover, further studies are required to elucidate the mechanism of how ALA application could regulate specific metabolic reactions to achieve enhanced resistance in seeds to temperature stress. Therefore, this study provides the first investigation ALA effects *E. nutans* on cold stress. Our specific objectives were: (1) to investigate whether ALA could improve *E. nutans* seed germination under low temperature and (2) to further explore the mechanism of exogenous ALA pre-soaking improving seed germination via determining antioxidant enzyme activities, lipid peroxidation, seed respiration rate, H^+^-ATPase activity and endogenous ALA concentration in *E. nutans* seeds under cold stress.

## Materials and Methods

### Plant Material and Treatments

*Elymus nutans* seeds were obtained from two sources: seeds of Damxung (DX) were collected in September 2012, from wild plants growing in Damxung County (30°28.535′N, 91°06.246′E, altitude 4678 m), located in the middle of Tibet, China. Agriculture and Animal Husbandry Bureau in Tibet responses for Damxung County. *E. nutans* occurs naturally and abundantly at altitudes between 3,000 and 5,000 m in the Qinghai-Tibetan Plateau, the field studies did not involve endangered or protected species. And Zhengdao (ZD) seeds were obtained in September 2012, from Beijing Rytway Ecotechnology Co., Ltd., located in Changping District (40°06.595′N, 116°24.383′E, altitude 550 m), Beijing, China. Seeds were cleaned and stored at 4°C in paper bags until the start of the experiments. In a preliminary experiment, two sources of *E. nutans*, DX and ZD, were found that ZD was more susceptible to cold stress than DX.

Seeds were surface sterilized in 1% (w/v) sodium hypochlorite for 10 min and rinsed several times with distilled water. Seeds were placed on double layers of filter paper wetted with 5 ml of 0, 0.1, 0.5, 1, 5, 10 or 25 mg l^−1^ ALA (Sigma Aldrich, St. Louis, MO, USA) solution in Petri dishes of 9 cm diameter. Seeds were kept at 25°C in the dark for 24 h [9]. Soaked seeds were then washed for 1 min under running water.

Preliminary investigation demonstrated inhibition of germination of *E. nutans* at 5°C compared to higher temperatures (10–30°C). After ALA application, germination tests were carried out in a plant growth chamber (Percival E-36L, Percival Scientific. Inc., USA) at a day/night temperature 5°C/5°C, a relative humidity of 70%, a day/night regime of 14 h/10 h and a photosynthetic photon flux density (PPFD) of 100 µmol m^−2 ^s^−1^. The lighting system is lit by (16) 17 W cool white fluorescent lamps and (2) 40 W incandescent lamps properly spaced for uniform light intensity. Fifty seeds were placed on two layers of filter paper moistened with 5 ml of distilled water in covered 9 cm Petri dishes. To prevent fungal contamination, 1 ml of 0.5% Captan was added. Petri dishes were arranged in completely randomized design with five replications. Seed germination was defined as root or shoot emergence [21]. Germination was recorded daily until the numbers stabilized (for 15 days). Dry weights of seeds and chlorophyll (Chl) contents of seedlings were measured following germination determination.

To further explore possible cold adaptation mechanisms of seed germination in *E. nutans* promoted by ALA treatment, a second batch from both sources remained untreated or was imbibed in ALA solution using previously described conditions at either 5°C or 25°C for 24 h. All seeds were germinated at 5°C for 5 d in the growth chamber using previously described temperature and light conditions. After 5 d of cold stress [22], the germinating seeds were used for further bio-chemical and physiological measurements. Seed treatments at 25°C was used as the control (and identical the rest of growth conditions).

### Assay of Dry Weight

Seeds (mainly endosperms and pericarps) were isolated and dried in oven at 80°C for 72 h and their dry weights were determined.

### Determination of Chlorophyll (Chl) Concentration

The concentration of Chl were determined spectrophotometrically using 80% acetone as a solvent [23]. Extract absorbance was measured at 645 and 663 nm with Optizen 5100 UV spectrophotometer (Shanghai, China).

### Analysis of Lipid Peroxidation

Membrane lipid peroxidation was measured as the concentration of malondialdehyde (MDA) produced using 10% (w/v) trichloroacetic acid (TCA), according to Dhindsa et al. [24]. The absorbance of the supernatant was measured at 450, 532, and 600 nm.

### Measurement of Hydrogen Peroxide and Superoxide Radical

Hydrogen peroxide concentration was measured according to Veljovic-Jovanovic et al. [25]. Seeds (0.5 g) were ground in liquid nitrogen and the powder was extracted in 2 ml 1 M HClO_4_ in the presence of 5% polyvinylpyrrolidone (PVP). The absorbance was read at 590 nm. Hydrogen peroxide concentration was calculated from a standard curve prepared in a similar way and expressed as nmol g^−1^ FW.

Superoxide radical production rate was determined by the modified method according to Elstner and Heupel [26]. Seeds (1.0 g) were homogenized in 3 ml 50 mM potassium phosphate buffer (pH 7.8) and centrifuged at 12,000×g for 20 min. The final solution was mixed with an equal volume of ethyl ether, and the absorbance of the pink phase was read at 530 nm.

### Quantification of Non-enzymatic Antioxidant Concentrations

Reduced glutathione (GSH) and oxidized glutathione (GSSG) concentrations were determined according to according to Law et al. [27] with some modifications. The germinating seeds (0.3 g) were homogenized with 5 ml of 10% (w/v) TCA and homogenate was centrifuged at 15,000×g for 15 min. To assay total glutathione, 150 ml supernatant was added to 100 ml of 6 mM 5,5′-dithiobis-(2-nitrobenzoic acid) (DTNB), 50 ml of glutathione reductase (10 units ml^−1^), and 700 ml 0.3 mM nicotinamide adenine dinucleotide phosphate (NADPH). The total glutathione content was calculated from the standard curve. All the reagents were prepared in 125 mM NaH_2_PO_4_ buffer, containing 6.3 mM ethylene diamine tetraacetic acid (EDTA), at pH 7.5. To measure GSSG, 120 ml of supernatant was added to 10 ml of 2-vinylpyridine followed by 20 ml of 50% (v/v) triethanolamine. The solution was vortex-mixed for 30 s and incubated at 25°C for 25 min. The mixture was assayed as mentioned above. Calibration curve was developed by using GSSG samples treated exactly as above and GSH was determined by subtracting GSSG from the total glutathione content.

After 0.2 g of germinating seeds was suspended in 3 ml of 6% TCA and was centrifuged at 4°C and 15,000×g for 20 min, the contents of AsA and total ascorbate were assayed at 525 nm [28]. The difference between the levels of total ascorbate and AsA was used for estimating the content of oxidized ascorbate.

### Assay of Antioxidant Enzymes

The germinating seeds (0.5 g) were homogenized with a mortar and pestle at 4°C in 5 ml 50 mM phosphate buffer (pH 7.8) containing 1 mM EDTA and 2% PVP. Homogenate was centrifuged at 12,000×g for 20 min at 4°C and the supernatant was used for enzyme activity assays. Protein content in the supernatant was determined according to the method of Bradford [29] with bovine serum albumin (BSA) as standard.

The assay for ascorbate peroxidase (APX) activity was measured in a reaction mixture of 3 ml containing 100 mM phosphate (pH 7), 0.1 mM EDTA-Na_2_, 0.3 mM ascorbic acid, 0.06 mM H_2_O_2_ and 100 µl enzyme extract. Change in absorption was observed at 290 nm 30 s after addition of H_2_O_2_ [30]. One unit of APX forms 1 µM of ascorbate oxidized per minute under assay conditions. Activity of catalase (CAT) was measured by following the consumption of H_2_O_2_ at 240 nm according to Cakmak and Marschner [31]. The decrease in the absorption was followed for 3 min and a breakdown of 1.0 µM H_2_O_2 _ml^−1 ^min^−1^ was defined as 1 Unit of CAT activity. Glutathione reductase (GR) activity was measured by following the decrease in absorbance at 340 nm due to NADPH oxidation. The reaction mixture contained tissue extract, 1 mM EDTA, 0.5 mM GSSG, 0.15 mM NADPH and 50 mM Tris–HCl buffer (pH 7.5) and 3 mM MgCl_2_ [32]. The reaction was started by using NADPH. Activity of superoxide dismutase (SOD) was determined according to Beauchamp and Fridovich [33] by following the photo-reduction of nitroblue tetrazolium (NBT) at 560 nm. One Unit of SOD activity was defined as the amount of enzyme required to cause a 50% inhibition of NBT reduction.

### Determination of Seed Respiration Rates

Respiration rates were measured according to Zheng et al. [34]. A closed gas collecting system was used to measure CO_2_ production during seed germination. After 5 d of cold stress, germinating seeds were sealed in an internally ventilated chamber with a volume of 0.2 L. The chamber was coupled with a GXH-3010F IRGA (infra-red gas analyzer, Huayun Instrument Research Institute Co., Beijing, China). Respiration rate of seed was calculated according to the slope of CO_2_ increase in the chamber.

### Assay of Seed Adenosine Triphosphate (ATP) content

The ATP content of seeds was determined by spectrofluorometry as described by Zheng et al. [34]. One g of germinating seeds were finely sliced and put into 5 ml acetone, and placed in boiling water bath for 5 min until the acetone fully evaporated. Three ml of 20 mM Tris–HCl buffer (pH 7.6) were added to the sample and heated in a boiled water bath for 10 min, and then immediately cooled down in an ice bath. The tubers was centrifuged at 3,000×g for 10 min, and the supernatant was collected. Bioluminescence produced by adding the ATP extract was measured with the ATP Bioluminescent Assay Kit (luciferin-luciferase reagent, the Detect Technical Institute, Shenzhen, China) using a SHG-D Bioluminescence and Chemiluminescence Meter (The Detect Technical Institute, Shenzhen, China).

### Assay of Plasma Membrane (PM) H^+^-ATPase Activity

Plasma membrane vesicles were isolated from germinating seeds by phase partitioning according to the procedure by Palmgren et al. [35]. Samples were ground in ice cold homogenization buffer containing 50 mM 3-(*N*-morpholino) propanesulphonic acid- Bis-tris Propane (MOPs–BTP) (pH 7.5), 330 mM sucrose, 5 mM EDTA, 5 mM dithiothreitol (DTT), 0.5 mM phenylmethanesulphonylfluoride, 0.2% (w/v) casein, 0.2% bovine serum albumin, and 0.5% PVP-40. Homogenate was filtered through four layers of cheesecloth and the filtrate was centrifuged at 10,000×g for 15 min at 4°C. Supernatant was collected and centrifuged at 80,000×g for 45 min, and the resulting precipitate was resuspended in buffer consisting of 330 mM sucrose, 5 mM potassium phosphate (pH 7.8), 5 mM KCl, 0.1 mM EDTA, and 1 mM DTT. Homogenate was loaded onto a two-phase system containing 6.5% Dextran T-500 (Sigma–Aldrich, USA), 6.5% (w/w) polyethylene glycol (PEG)-3350 (Sigma–Aldrich), 250 mM sucrose, 5 mM KH_2_PO_4_ (pH 7.8), 4 mM KCl, and sterile distilled water. After the batch procedure, the resulting upper phase was mixed with a dilution buffer consisting of 5 mM MOPs–BTP (pH 7.5), 330 mM sucrose, and 5 mM KCl, and was centrifuged at 100,000×g for 60 min. PM vesicles obtained were either used immediately or stored at −80°C, pending analysis.

PM H^+^-ATPase activity was measured according to the procedure of Ahn et al. [36]. PM H^+^-ATPase activity was measured with 5 µg protein in 0.5 ml of reaction solution that contained 30 mM MOPs–BTP (pH 6.5), 3 mM MgSO_4_, 50 mM KCl, 1.5 mM ATP, and 0.05% Triton-X100. After 30 min at 37°C, the reaction was stopped by adding 500 µl of 5% trichloroacetic acid, 2 ml of 100 mM sodium acetate, 300 µl of 1% ascorbic acid, 60 µl of 10 µM CuSO_4_, and 300 µl of 1% ammonium molybdate in 0.025 mM H_2_SO_4_. Following additional 10 min at 30°C, absorbance at 720 nm was measured with an Optizen 5100 UV spectrophotometer (Shanghai, China). Difference between samples with and without 0.1 mM vanadate, which is a specific PM H^+^-ATPase inhibitor, was expressed as the PM H^+^-ATPase activity. A standard curve of phosphate in the reaction mixture was included for each assay.

### Determination of ALA Concentration

Germinating seeds (0.1 g) were homogenized in 5 ml of 1 M sodium acetate buffer (pH 4.6) and centrifuged at 12,000×g for 10 min. The assay mixture consisted of 0.1 ml of supernatant, 0.4 ml of distilled water, and 25 µl of acetylacetone. The assay medium was mixed and heated in a boiling water bath for 10 min. The extract was then cooled at room temperature, and an equal volume of modified Ehrlich’s reagent was added and vortexed for 2 min. After 10 min of incubation, absorbance of the extract was measured at 555 nm and ALA concentration was determined from the standard curve of ALA [37].

### Statistical Analysis

Each experiment was repeated three times. All values were expressed as means ± SD. Statistical analyses were performed by analysis of variance (ANOVA) using SPSS-17 statistical software (SPSS Inc., Chicago, IL, USA). Means were separated using Duncan’s least significance difference test at *P<*0.05.

## Results

### Effect of ALA on Seed Germination, Dry Weight and Chl Content

**Figure 1:**
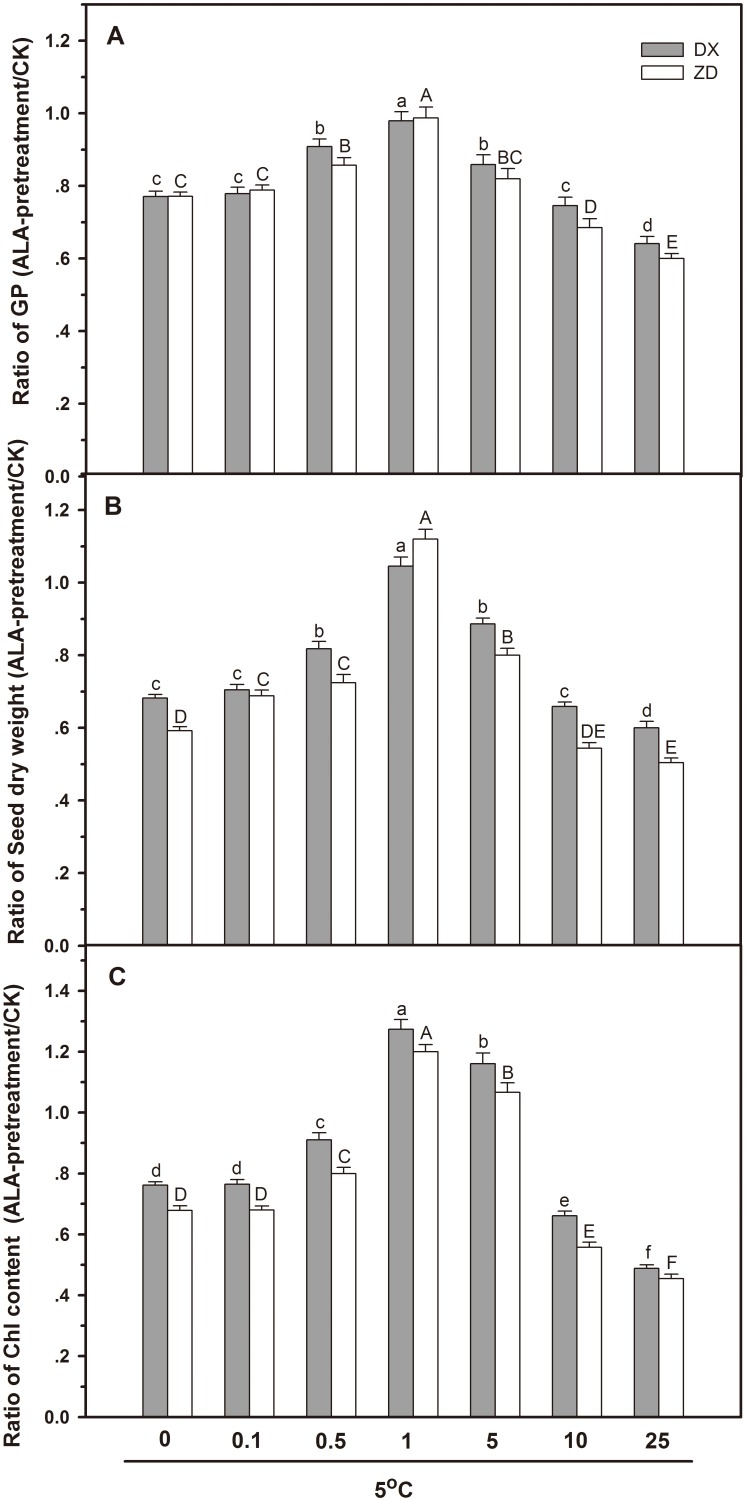
Effect of ALA applications on the ratio of germination percentage (GP) (A), dry weights of seed (B), and chlorophyll (Chl) concentration (C) under cold stress in *E. nutans* (DX, Damxung and ZD, Zhengdao). ALA pretreatment at different concentrations (0, 0.1, 0.5, 1, 5, 10, and 25 mg l^−1^) were carried out prior to cold stress (5°C). Bars represent the mean ± SD (n = 3). Bars with different letters are significantly different at the 5% level.

ALA concentrations ranging from 0 to 25 mg l^−1^ were applied to *E. nutans* seeds to investigate the response for cold resistance. The ratio of germination percentage (GP) was improved for seeds from both sources when pre-treated with 0.5, 1 or 5 mg l^−1^ ALA. However, ALA concentrations above 1 mg l^−1^ caused the ratio of GP reduction in a dose dependent manner (Fig. 1A). On the other hand, 1 mg l^−1^ ALA alone did not have any effect on the ratio of GP compared to the unsoaked (data not shown).

The ratio of dry weight of DX and ZD increased with the concentrations of ALA, peaking at 1 mg l^−1^ ALA prior to cold stress, and decreased at 5 mg l^−1^ ALA. These concentrations alone did not alter dry weight compared to controls (data not shown). Treatment with 10 or 25 mg l^−1^ ALA resulted in a major seed dry weight loss compared to the unsoaked (Fig. 1B).

Pre-treatment with low ALA concentrations (0.1–5 mg l^−1^) prevented the loss of Chl content in both sources of *E. nutans* seed, whereas exposure to higher ALA concentrations (10 and 25 mg l^−1^) brought about a dose dependent decrease, reaching a minimum in DX and ZD pre-treated with 25 mg l^−1^ ALA compared to the untreated (Fig. 1C).

ALA was effective in enhancing cold resistance in *E. nutans* seeds and seedlings up to 5 mg l^−1^. The best results were obtained in seeds pre-treated with 1 mg l^−1^ ALA. As a result, 1 mg l^−1^ ALA was applied in subsequent experiments.

### Effect of ALA on MDA, H_2_O_2_ Concentrations and O_2_^•−^ Level

**Figure 2:**
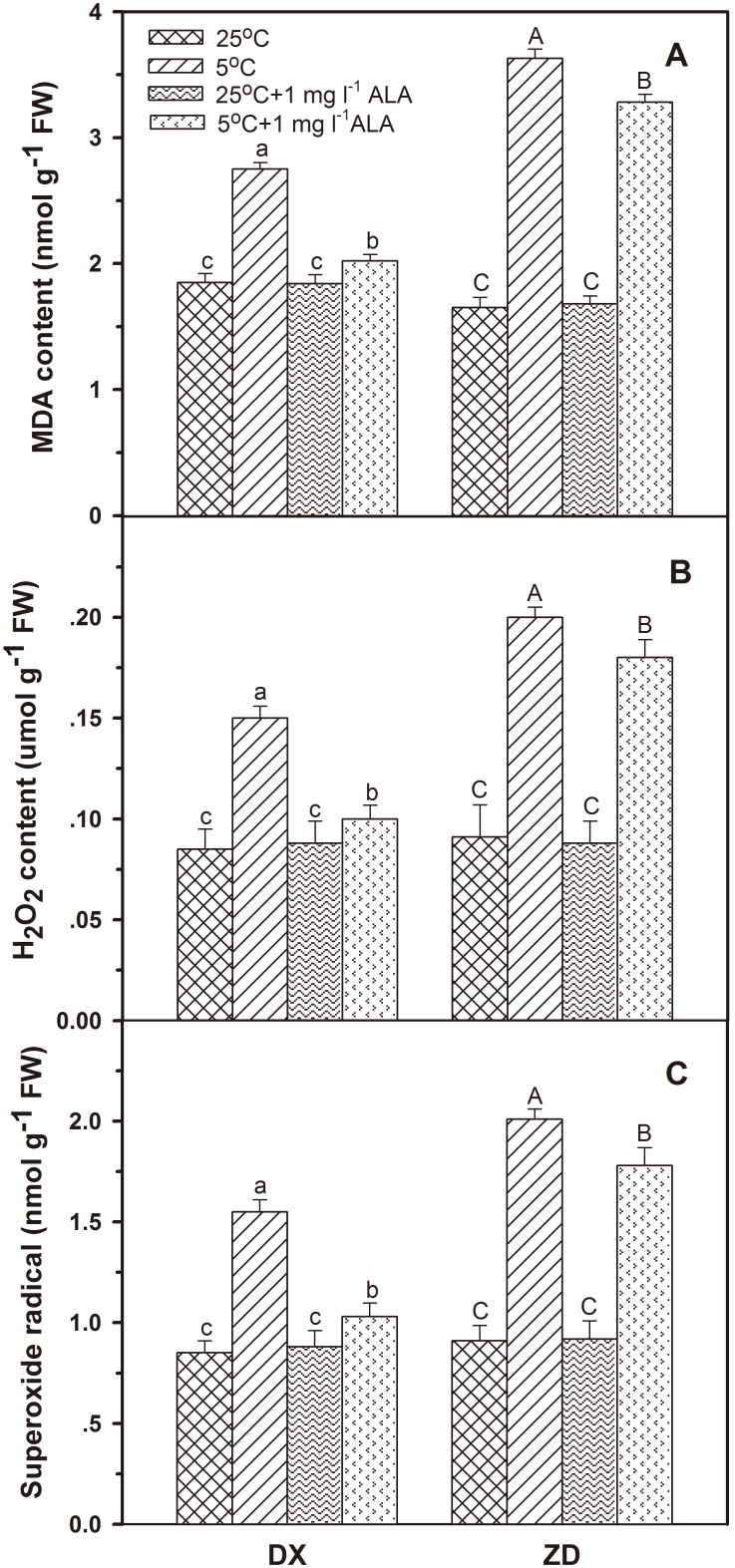
Effect of ALA applications on MDA (A), H_2_O_2_ concentrations (B) and the rate of O_2_^•−^ generation (C) under cold stress in *E. nutans* (DX, Damxung and ZD, Zhengdao). Bars represent the mean ± SD (n = 3). Bars with different letters are significantly different at the 5% level.

Contents of MDA and H_2_O_2_, and release rate of O_2_^•−^ in both sources of *E. nutans* seed increased after exposure to cold stress for 5 d (Fig. 2). Cold treatment increased (*P<*0.05) MDA contents by 48.6% and 120.0% in seeds of DX and ZD, respectively. DX seeds exhibited a 76.5% and 119.7% increase in levels of H_2_O_2_ and O_2_^•−^, while a 82.4% and 120.9% increase was observed in ZD under cold stress. Exogenous ALA treatment alleviated (*P*<0.05) the cold induced accumulation of MDA and H_2_O_2_, and decreased (*P*<0.05) the release rate of O_2_^•−^ in germinating *E. nutans* seeds. Under normal condition, pretreatment of seeds with 1 mg l^−1^ ALA did not significantly change MDA, H_2_O_2_ concentrations and O_2_^•−^ generation in seed from either source.

### Effect of ALA on Concentrations of Non-enzymatic Antioxidants

**Figure 3:**
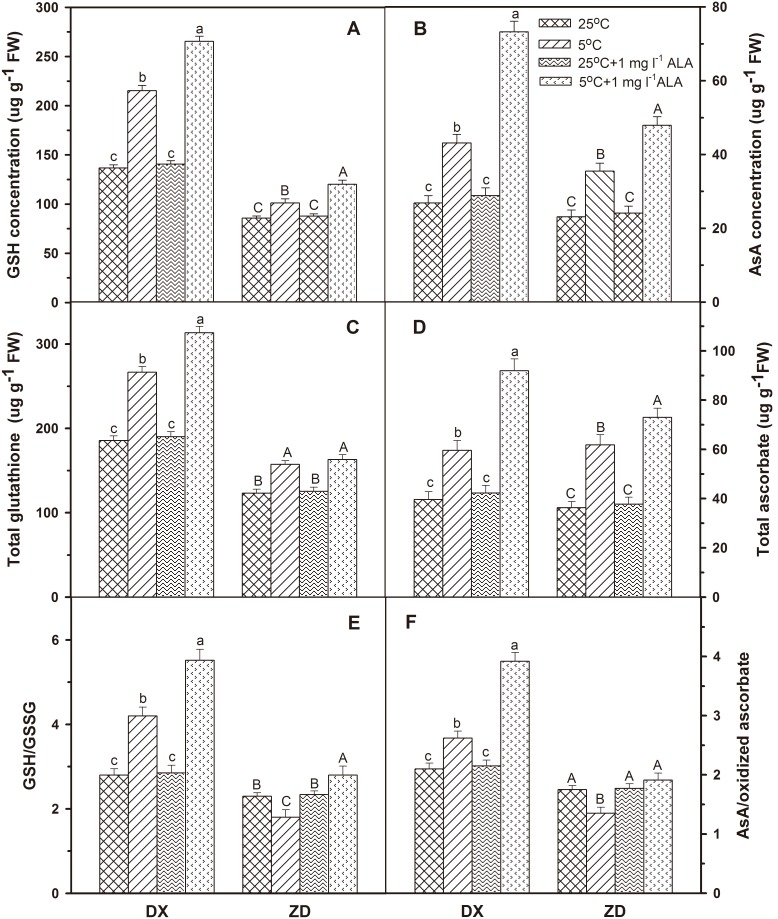
Effect of ALA applications on GSH (A), AsA (B), total glutathione (C), total ascorbate concentrations (D), and the ratios of GSH/GSSG (E) and AsA/oxidized ascorbate (F) under cold stress in *E. nutans* (DX, Damxung and ZD, Zhengdao). Bars represent the mean ± SD (n = 3). Bars with different letters are significantly different at the 5% level.

The data regarding increased glutathione (GSH), ascorbic acid (AsA), total glutathione, total ascorbate concentrations, and the ratios of reduced/oxidized glutathione (GSH/GSSG) and reduced/oxidized ascorbate (AsA/oxidized ascorbate) in DX seeds under cold stress had been shown in Fig. 3, while the ratio of GSH/GSSG and AsA/oxidized ascorbate decreased in ZD (*P*<0.05). ALA at lower concentration 1 mg l^−1^ showed improvement in GSH, AsA, total glutathione and ascorbate concentrations, and the ratio of GSH/GSSG and AsA/oxidized ascorbate in germinating *E. nutans* seeds under cold stress. Antioxidants showed no significant changes when seeds were treated with ALA alone.

### Effect of ALA on Activities of Antioxidant Enzymes

**Figure 4:**
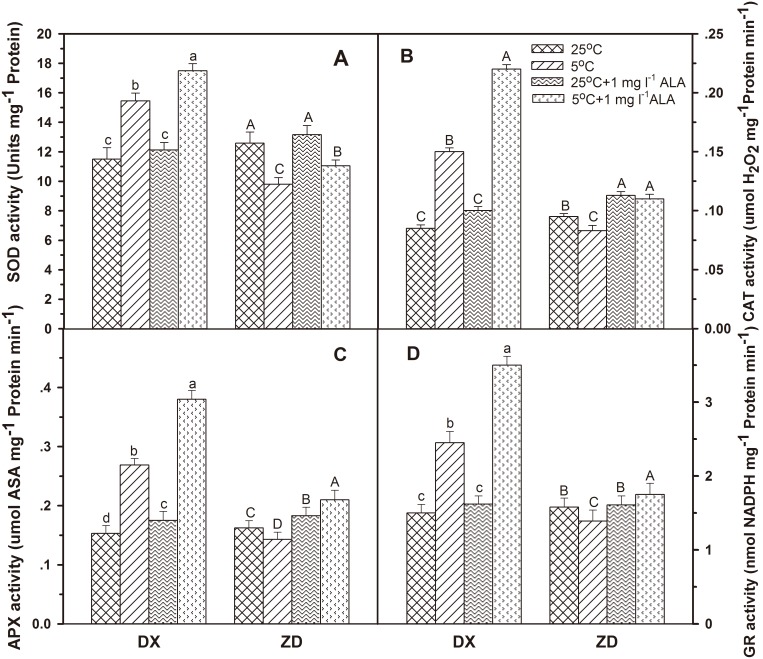
Effect of ALA applications on the activities of SOD (A), CAT (B), APX (C), and GR (D) under cold stress in *E. nutans* (DX, Damxung and ZD, Zhengdao). Bars represent the mean ± SD (n = 3). Bars with different letters are significantly different at the 5% level.

Activities of SOD, CAT, APX and GR increased (*P*<0.05) by 34.3%, 76.5%, 75.4%, 63.3% in DX seeds, but decreased (*P*<0.05) by 22.1%, 12.6%, 11.9%, 12.1% in ZD seeds subjected only to cold treatment, respectively (Fig. 4). Pretreatment with ALA increased SOD, CAT, APX and GR activities (*P*<0.05), especially in DX seeds. Under control conditions, activities of all four antioxidant enzymes were not significantly influenced by exogenous ALA.

### Effect of ALA on Seed Respiration Rate and ATP Content

**Figure 5:**
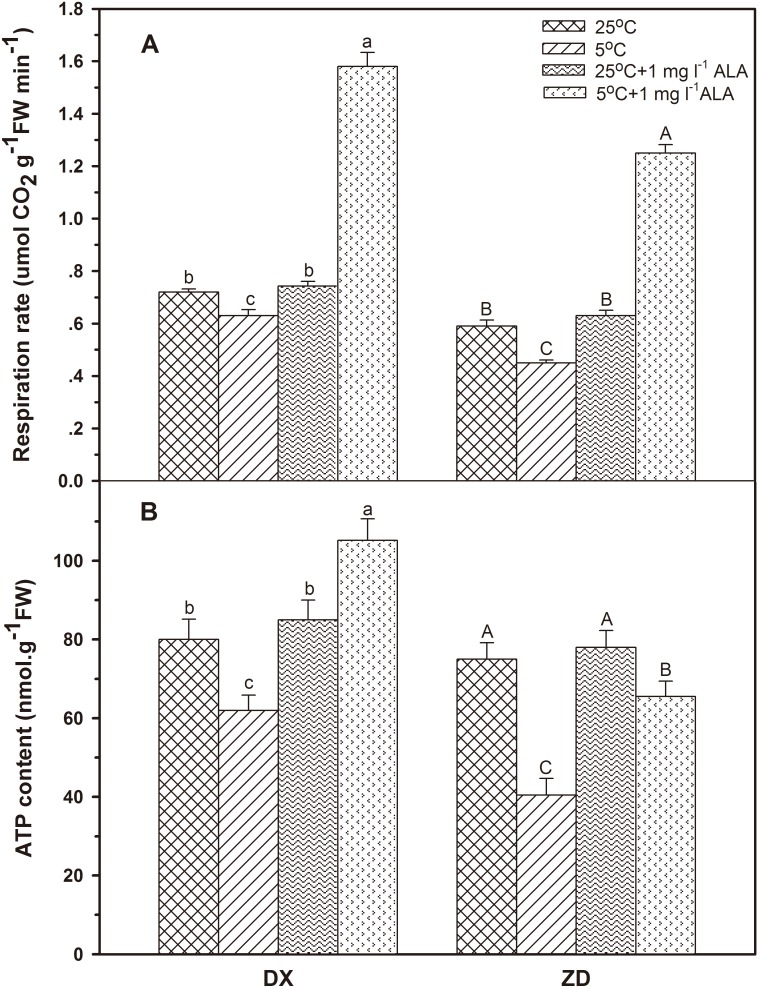
Effect of ALA applications on seed respiration rate (A) and ATP content (B) under cold stress in *E. nutans* (DX, Damxung and ZD, Zhengdao). Bars represent the mean ± SD (n = 3). Bars with different letters are significantly different at the 5% level.

After 5 d of cold stress, seed respiration rate sharply decreased in seed from *E. nutans* sources (Fig. 5A). Compared to control, exogenous ALA treatment increased the respiration rate in both seed sources of *E. nutans*. Treatment with ALA, under normal conditions, had no effect on seed respiration rate compared with control. A similar pattern of changes in germinating seed ATP content in response to exogenous ALA was observed in *E. nutans* under cold stress (Fig. 5B). Exogenous ALA treatment increased seed ATP content in seeds. ATP content in ALA soaked DX and ZD seeds increased 83.1% and 61.7% after 5 d stress compared to non-ALA treatment.

### Effect of ALA on Activities of PM H^+^-ATPase

**Figure 6:**
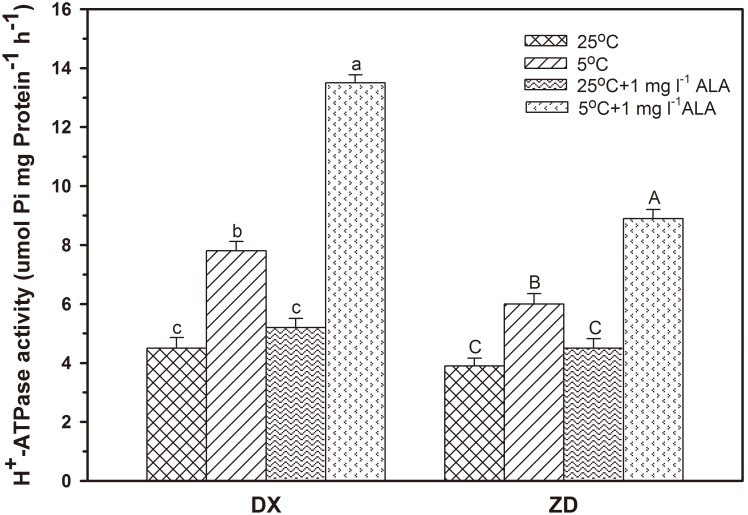
Effect of ALA applications on the activities of PM H^+^-ATPase under cold stress in *E. nutans* (DX, Damxung and ZD, Zhengdao). Bars represent the mean ± SD (n = 3). Bars with different letters are significantly different at the 5% level.

Cold stress resulted in a 73.3% and 53.8% increase (*P*<0.05) in H^+^-ATPase activity in DX and ZD, compared with control (Fig. 6). Exogenous ALA treatment prior to cold stress further enhanced (*P*<0.05) the activities of H^+^-ATPase in both seeds. In contrast, enhancement of the activities of H^+^-ATPase did not occur in seeds pretreated with ALA alone.

### Endogenous ALA Production

**Figure 7:**
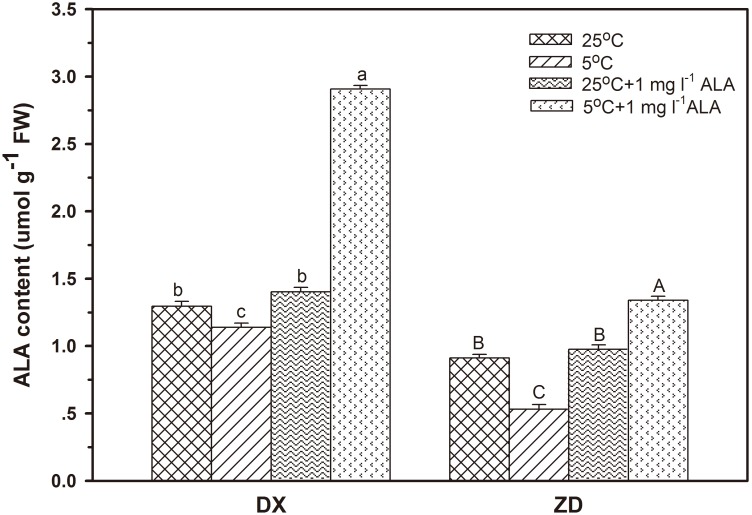
Effect of ALA applications on endogenous ALA concentrations under cold stress in *E. nutans* (DX, Damxung and ZD, Zhengdao). Bars represent the mean ± SD (n = 3). Bars with different letters are significantly different at the 5% level.

To verify the protective effect of exogenous ALA applied to seeds under cold stress, endogenous ALA release rates were measured. Endogenous ALA release rates decreased (*P*<0.05) after 5 d of cold stress. Under cold stress, pretreatment with 1 mg l^−1^ ALA increased (*P*<0.05) endogenous ALA release in both seeds, especially in DX throughout the stress period. Application of 1 mg l^−1^ ALA alone showed no change in endogenous ALA in both seeds (Fig. 7).

## Discussion

Cold stress can lead to biochemical and physiological changes in plant tissues. Inhibitory effects of cold stress on *E. nutans* seed germination is consistent with earlier reports of low temperature stress in pepper seed [2]. In the present study, cold stress significantly reduced seed GP and dry weight for both seed sources. Under cold stress, cold-resistant DX showed significantly greater GP and seed dry weight than cold-sensitive ZD. Observed reduction of GP and dry weight in both seeds might be due to oxidative stress induced by cold [38]. Treatment with ALA significantly enhanced GP and dry weight for sources of both *E. nutans* seeds. Pre-soaking with ALA may have potential to enhance stress resistance by decreasing the lipid peroxidation [39] by activating the heme-based antioxidant enzyme systems to scavenge ROS like H_2_O_2_ [40].

Membranes are most susceptible to damage resulting from low temperature [41]. MDA is considered a final decomposition product of polyunsaturated fatty acids and it is used to determine oxidative damage [42]. In this study, MDA contents were increased after 5 d of cold stress. ALA alone had no effect on MDA level, but application of ALA under cold stress decreased MDA contents in germinating seeds, paralleling findings of Naeem et al. [43] who observed that ALA reduced MDA content under salinity stress in *Brassica napus*. Oxidative stress is increased by increases in ROS in cells under low temperature [7]. Observed increases in ROS level due to cold stress are similar to the findings of Zhang et al. [13]. In our study, ALA significantly reduced production of ROS under cold stress, suggesting that ALA can improve plant resistance to oxidative stress. Ali et al. [16] reported that application of ALA at 25 mg l^−1^ concentration facilitated Cd stressed plants to detoxify the ROS using the antioxidant enzyme in *B. napus*.

ALA alleviates the membrane peroxidation resulting from ROS produced under stress conditions through different metabolism, and antioxidant capacity modulation was reported to be one of important pathways in many investigations [15], [16], [44]. GSH is an important component of the antioxidant system that scavenges ROS either directly or indirectly by participating in the ascorbate–glutathione cycle [45]. The key role of GSH in the antioxidant defense system is due to its ability to regenerate ascorbate (AsA) through reduction of dehydroascorbate via the ascorbate–glutathione cycle [46]. The high concentrations of AsA and GSH play roles in alleviating the injury caused by ROS [47]. ALA application at low concentrations can enhance the GSH contents in the roots of *B. napus* [16]. In the presence of cold stress, GSH, AsA, total glutathione, total ascorbate concentrations, and the ratios of GSH/GSSG and AsA/oxidized ascorbate increased significantly when applied with ALA. Similarly, Liu et al. [48] reported that PEG treatment increases the concentrations of GSH and AsA and the ratios of AsA/oxidized ascorbate and GSH/GSSG, as well as decreases ROS level. ALA pretreatment can induce the synthesis of heme-based molecules [49]. ROS induction under cold stress may be due to the reason that ALA is a precursor of heme biosynthesis, so it can boast up the activities of heme-based molecules and can help in scavenging the ROS under cold conditions [16].

Antioxidant enzymes are considered to be the most efficient mechanisms against oxidative stress. When exposed to oxidative stress, the synthesis and activity of antioxidant enzymes are increased [50]. Among these enzymes, SOD is a major scavenger of O_2_^•−^, catalyzing the dismutation of superoxide radicals to H_2_O_2_ and O_2_. CAT directly scavenge H_2_O_2_, while APX and GR are involved in the AsA–GSH cycle, a non-enzymatic pathway that removes O_2_^•−^ and H_2_O_2_ [44], [51]. The enhancement of the activities of antioxidant enzymes suggests that ROS induced these changes in different cellular compartments [52]. In this study, DX is more resistant to cold because antioxidant enzyme activities to remove newly produced ROS are higher. Under cold stress, a greater increase in antioxidant enzyme activities and lower levels of H_2_O_2_ and O_2_^•−^ were found in DX than in ZD. ALA has been reported to stimulate the activities of antioxidative enzymes under stress conditions [3]. Our results showed treatment with ALA further enhanced those antioxidant enzyme activities under cold stress. Similar observation were made by Zhang et al. [13] in cucumber. Thus, ALA contributed to reduce oxidative stress via higher antioxidant concentrations and antioxidant enzyme activities in germinating *E. nutans* seeds, thereby improving germination percentages under cold stress.

Seed treatment with ALA is known to improve GP and physiological processes under various stress conditions [10]. Similar observation had shown that ALA as a pre-soaking seed treatment improved the low-temperature resistance of pepper (*Capsicum annuum*) by enhancing final GP and germination rate [2]. Our results indicated that the treatment with ALA significantly enhanced GP for both sources of *E. nutans* seeds. Likewise, application of ALA treatment increased the seed germination of pakchoi (*Brassica campestris*), which was due to the improved seed respiration rate under salt stress [53]. The results were consistent with our finding that ALA treatment increased seed respiration rate in both sources of *E. nutans* seeds. Seed priming techniques have been used to increase germination and improve activities antioxidant enzyme by plant hormones/regulators under different various stress conditions [54], [55]. In pepper, a remarkable enhancement in GP was observed through seed priming with ALA under cold stress [2]. The improved GP observed in our study is most likely due to the enhanced antioxidant enzymes activities just like ALA improved GP in pepper [2], [3]. Therefore, ALA may be employed as effective approach to improving seed germination and plant growth under stress conditions.

In the plant mitochondria, electron transfer along the respiration chain is coupled to the formation of ATP [56], and the redundant electron leads to the formation of ROS if ATP synthesis is blocked [57]. ALA is the first precursor in the biosynthesis of porphyrin compounds such as chlorophyll and heme, a key element required for cytochrome c activity in the respiration chain of the mitochondrion [10]. Respiration, a temperature-dependent and heme-requiring process, increases during germination in order to provide necessary energy. Under cold stress, decreased respiration rates and ATP contents were observed in germinating seeds from both sources, while respiration was enhanced in seeds treated with ALA. Thus, it is suggested that exogenous ALA may promote ATP synthesis and enhance seed activity (respiration rate), both having a positive effect on seed germination under cold stress. A similar antioxidant stress effect of exogenous ALA was observed in salt-stressed pakchoi seeds [53]. In addition, cold stress reduced Chl concentration and endogenous ALA level while application of exogenous ALA increased Chl concentration and endogenous ALA release in germinating seeds, suggesting application of exogenous ALA prior to cold stress could mitigate inadequate biosynthesis problem [3].

PM H^+^-ATPase plays a role in the adaptation of plants to stress conditions. An increase in permeability related to membrane damage and a change in its viscosity and fluidity are observed in plants that have been subjected to low temperature [41]. Increased generation of a proton gradient by PM H^+^-ATPase is required to maintain ionic balance and replenish the loss of organic compounds [58]. In this study, the activity of PM H^+^-ATPase increased after 5 d of cold stress in germinating seeds, agreeing with reports of Kim et al. [59] in cold treated camelina and rapeseed. Pretreatment with ALA further elevated H^+^-ATPase activity compared with cold stress alone, which might indicate ALA acts as a signaling molecule, inducing increases in the activities of H^+^-ATPase.

In conclusion, results of our study revealed that pre-soaking with 1 mg l^−1^ ALA improved *E. nutans* germination compared with non-ALA treated seeds. Protective effects of ALA on germinating seeds result from stimulation of activities of heme-based non-enzymatic antioxidants and antioxidant enzymes which help in scavenging the ROS under cold stress, especially in cold resistant DX seeds. In addition, ALA might act as a signaling molecule, inducing increased H^+^-ATPase activity and promoting ATP synthesis. Our finding that ALA could be used as a seed treatment to enhance GP in *E. nutans* under cold stress may be useful in helping to solve serious problems occurring on a global scale due to low temperatures.
